# Association of antihypertensive drugs, intestinal ischemia, and breast diseases: A drug-target Mendelian Randomization study

**DOI:** 10.1097/MD.0000000000049141

**Published:** 2026-06-05

**Authors:** Jiaxuan Li, Yiqun Ma, Kaili Zhang

**Affiliations:** a The Second Hospital of Hebei Medical University, Shijiazhuang, People’s Republic of China; b The Second Affiliated Hospital of Kunming Medical University, Kunming, People’s Republic of China.

**Keywords:** antihypertensive drugs, breast disease, drug-targeting Mendelian Randomization, intestinal ischemia, mediating effect

## Abstract

Both hypertensive and breast diseases impose a significant societal burden. However, the correlation between antihypertensive drug use and breast cancer remains controversial. We aimed to determine their potential correlations and explore their possible mechanisms. We performed summary data-based Mendelian Randomization (SMR) analysis of expression quantitative trait loci (eQTL) data from the blood of patients with breast diseases. We then performed drug-targeting Mendelian Randomization (MR) analysis using blood pressure-related single nucleotide polymorphism (SNPs) as instrumental variables (IVs). False discovery rate (FDR) correction was performed for reliable results, and colocalization analysis was performed to clarify shared gene variants. Mediation MR methods were used to investigate the mechanistic pathways involved in this association. Combined with the results of SMR analysis and drug-targeting MR analysis, loop diuretics aggravated overall breast cancer (OR_SMR = 0.842, 95% CI_SMR: 0.778–0.911, P_SMR_FDR = 0.008) and ER + breast cancer (OR_SMR = 0.854, 95% CI_SMR: 0.778–0.937, P_SMR_FDR = 0.008) within SLC12A2 gene loci. Colocalization analysis showed that SLC12A2-targeted drugs share a common variant site with breast cancer. Mediation analyses suggested that the pathway related to intestinal ischemia mediated 73.711% of the total effect on over breast cancer risk in the SLC12A2 gene loci. Antihypertensive drugs increase the overall risk of breast cancer and ER + breast cancer within the SLC12A2 gene. And overall breast cancer risk which may result from intestinal ischemia. Given the long-term safety of antihypertensive drugs, significance should be attached to the prevention and early detection of breast cancer in patients taking loop diuretics.

## 1. Introduction

Diverse breast diseases are highly prevalent and have emerged as significant threats to women health.^[1]^ There are 5 major categories of breast cancer: inflammatory breast disorders, breast hyperplasia, benign breast neoplasms, breast cysts, and breast cancer. Specifically, 2.26 million new breast cancer patients were diagnosed globally between 2010–2019, ranking second in incidence among all cancers in women, causing 680,000 deaths.^[2]^ According to current investigations, early detection and self-care of breast disease can effectively reduce its incidence and mortality rates of breast disease.^[3]^ Hence, it is imminent to determine the unrecognized risk and protective factors for breast diseases.

Hypertension is a serious public health problem, and nearly 47.7% of adults will suffer from hypertension from 2021 to 2023.^[4]^ Additionally, hypertension is strongly correlated with heart failure, kidney disease and cancer.^[5,6]^ Because of the high prevalence and mortality rates of the 2 illnesses, many studies are currently focusing on the relationship between hypertension, use of antihypertensive drugs, and breast diseases. Hypertension may promote cancer through systematic inflammatory processes, modulation of associated cytokines, and alteration of the cancer microenvironment.^[7,8]^

The relationship between antihypertensive drug use and the risk of breast cancer has attracted the attention of an increasing number of scholars, but the findings are not fully consistent. De Miranda et al revealed that angiotensin-converting enzyme inhibitors (ACEIs) and angiotensin II receptor blockers (ARBs) can inhibit breast cancer progression by suppressing metastasis, proliferation, and angiogenesis.^[9]^ A review of 57 articles also suggested that the long-term use of ACEIs and ARBs was associated with reduced breast cancer risk, but the use of calcium channel blockers (CCBs), beta blockers (BBs), and diuretics could increase breast cancer risk.^[10]^ Moreover, 2 case-control studies demonstrated that CCBs, BBs, and diuretics increased the risk of breast cancer, but the role of ACEIs and ARBs was not significant.^[11,12]^ However, evidence from cross-sectional and cohort studies has suggested the absence of a significant relationship between antihypertensive medications (such as ACEIs, BBs, CCBs, and diuretics) and breast cancer development.^[13,14]^

Furthermore, Gollapudi et al found that 57.9% of patients hospitalized with chronic intestinal ischemia also had hypertension.^[15]^ Intestinal ischemia results in damage to the intestinal barrier, permitting an imbalance in intestinal microbiota, which promotes the onset of malignant and inflammatory diseases.^[16,17]^ However, only a few studies have demonstrated a correlation between these diseases.

Current research conclusions primarily drawn from observational studies. However, observational findings have several bias: confounding bias: potential confounding factors may simultaneously result in exposure and outcome diseases, and potential confounders may have led to conflicting results from previous observational studies; reverse causality: it is difficult to confirm causal relationships because of the contribution of reverse causation; measurement error and information bias, such as recall bias among study subjects. clinical drug trials are limited by funding constraints and ethical procedures. These factors compromise the accuracy of observational findings.

To fill this gap, drug-target Mendelian randomization (MR) analysis was used to provide more robust evidence of causality. MR analysis is based on the random allocation of parental alleles to offspring, wherein the use of genetic variants (usually single nucleotide polymorphism (SNPs)) as instrumental variables (IVs) effectively emulates the randomization process.^[18]^ In this study, the drug-target MR method was used. The potential implications for causal inference suggest that genetic variation within genes encoding drug targets contributes to the modulation of drug target activity and the corresponding phenotypic outcomes. This inference was made using genome-wide association studies (GWAS) and expression quantitative trait loci (eQTL) data as proxies for exposure.^[19]^ And MR analysis addresses observational research limitations: using SNP as IVs for exposure substantially mitigates confounding bias by eliminating environmental influences; randomization principle: the principle of genetic inheritance is similar to randomized controlled trials.; bidirectional MR analysis effectively excluded reverse causality; genetic variation, being innate, is less susceptible to environmental influences, recall error, or reporting biases.Therefore, drug-targeted MR methods have been used to achieve more efficient drug development and remove translation barriers. The mediation MR method was also incorporated into this process. According to previous studies, blood pressure (BP) is strongly associated with altered vascular status, whereas breast cancer is also associated with ischemia.^[20]^ Therefore, we further investigated the causal relationship between BP, intestinal ischemia, and breast cancer using mediated MR analysis.^[21]^

Using genetic proxies for drug targets, we conducted a drug-target MR study supplemented by colocalization analysis to determine the correlation between various antihypertensive drugs and the onset of breast diseases. This study identified new disease-associated genes that prevent the onset of breast diseases, facilitating primary prevention and increasing awareness among at-risk populations. As a result, the quality of life of patients with breast diseases can be improved. In addition, mediation analyses were performed from a genetic perspective on the causal relationships between hypertension, intestinal ischemia, and breast disease.

## 2. Materials and methods

### 2.1. Study design

**Figure 1.**
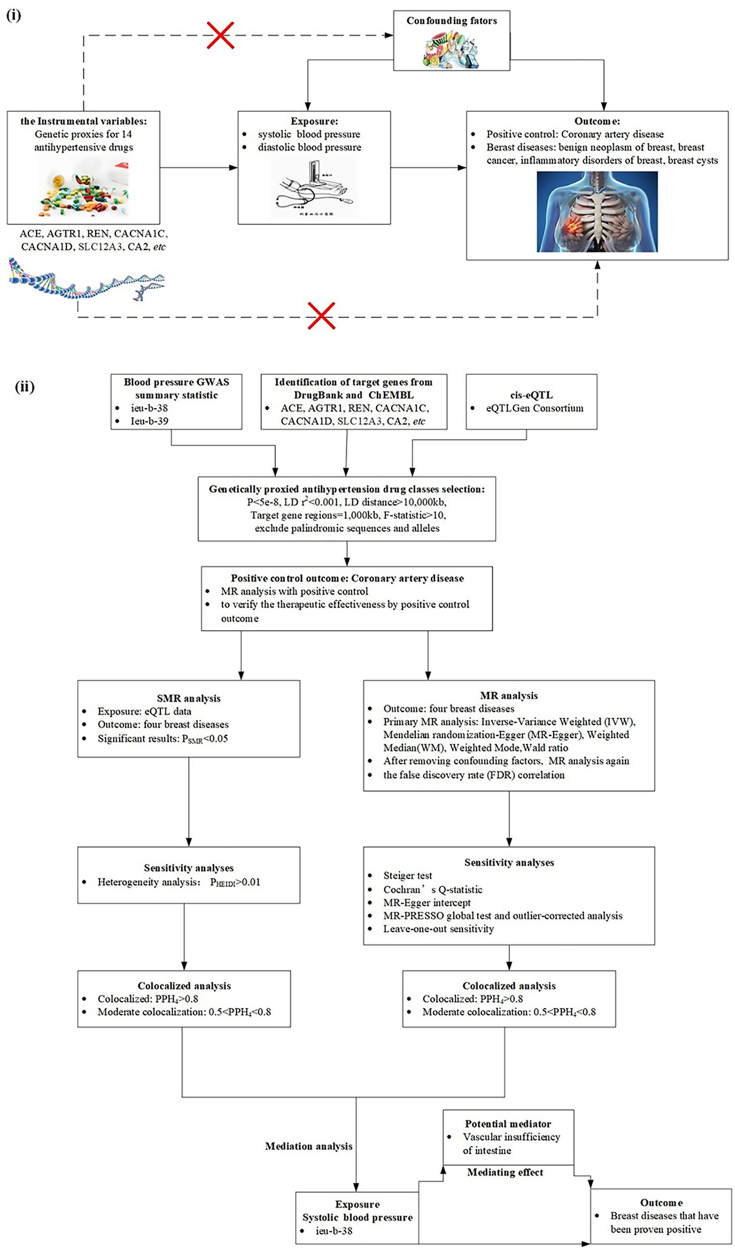
The overall design identifying the causal impact of antihypertensive drug targets on 4 breast diseases. The schematic images in the flowchart are sourced from the internet.

We utilized the GWAS summary and eQTLs data from the European population. Breast diseases include benign breast neoplasms, inflammatory breast diseases, and breast cysts.^[22]^ The validity of a 2-sample MR study is contingent upon 3 basic assumptions: Association: the IVs selected from the data demonstrate a strong correlation with the exposure; Independence: the IVs have been shown not to be linked to any confounders of the exposure; and Exclusivity: the IVs are correlated with the outcome solely through the exposure. The overall design of our study is shown in Figure 1, and the STROBE-MR checklist is shown in Table S1.^[23]^

### 2.2. Identification of antihypertensive drug targets

According to widely accepted guidelines, antihypertensive drugs are categorized as ACEIs, ARBs, BBs, dihydropyridines‌, non-dihydropyridines, thiazide diuretics (TDs), loop diuretics, potassium-sparing diuretics, renin inhibitors, mineralocorticoid receptor antagonists, alpha-1 blockers, centrally acting drugs, vasodilators, and angiotensin receptor-neprilysin inhibitors.^[24]^ Subsequently, the shared gene targets of the 14 antihypertensive drugs were identified using DrugBank (https://go.drugbank.com/) and ChEMBL (https://www.ebi.ac.uk/chembl/). We then retrieved the details of these genes from the NCBI Gene database (https://www.ncbi.nlm.nih.gov/), which are listed in Table S2.

### 2.3. Identification of BP

In this study, the downstream effects of pharmacological interventions (e.g., BP) were selected to model drug exposure. Genetic association data for systolic blood pressure (SBP) were derived from the IEU Open GWAS Project database (https://gwas.mrcieu.ac.uk/; Dataset: ieu-b-38). The present study included 757,601 European pedigree individuals from the UK Biobank and the International Consortium of Blood Pressure.^[25]^ Data on diastolic blood pressure (DBP) from the same population were also obtained (dataset: ieu-b-39),^[25]^ and all participants were adjusted for age, sex, and BMI.

### 2.4. Identification of gene expression data in the blood

Currently, it is widely accepted that peripheral blood is readily accessible for detection, and eQTL effects in peripheral blood may reflect those in other relevant tissues. As a result, eQTL data were obtained, encompassing a total of 31,684 blood samples from individuals of European descent, and provided transcriptomic profiles of 16,987 genes. Association analysis has also been used to identify loci of variation linked to gene expression.^[26]^ In addition, aggregated data for eQTL were extracted from the eQTLGen consortium (https://www.eqtlgen.org/).

### 2.5. Determination of study outcomes

Summary-level GWAS data related to benign breast neoplasms and inflammatory breast disorders were obtained from the FinnGen study (https://www.finngen.fi/en). Summary-level data of breast cancer, which combined GWAS meta-analysis data, iCOGS and Oncoarray, were obtained from the Breast Cancer Association Consortium (BCAC) database (https://bcac.ccge.medschl.cam.ac.uk).^[27]^ Data on breast cysts were obtained from the UK Biobank Project (http://www.nealelab.is/uk-biobank/). All participants were European populations, and no overlap existed between the populations in the exposure and outcome datasets. Details of the specific datasets are presented in Table S2.

### 2.6. Positive control

To verify the validity of the genetic targets, positive control analysis was performed. Antihypertensive drugs have been widely utilized to manage coronary artery disease (CAD); therefore, we obtained a CAD dataset from the GWAS catalog database (https://www.ebi.ac.uk/gwas/home) for analysis (Dataset: ebi-a-GCST005195).^[28]^ This is the largest dataset for CAD studies, with 122,733 cases and 424,528 controls. In this process, a 2-sample MR analysis was completed within the scope of the previously identified target gene.

### 2.7. SMR analysis

SMR analysis was conducted to ascertain the genetic impact of eQTL, which utilized summary data and followed the principles of MR. Horizontal pleiotropy was assessed using the heterogeneity in dependent instrument (HEIDI) method. The threshold of *P* value was 0.05 after false discovery rate (FDR) correlation.^[26]^

### 2.8. Two-sample MR analysis

We performed a positive control MR analysis, with SBP and DBP as exposures and CAD as outcomes. We then conducted a 2-sample MR analysis, with SBP and DBP as exposure and breast diseases as outcomes. First, we selected IVs with the following screening criteria: 1,000kb gene region cis-SNPs located near the drug target genes; SNPs strongly associated with BP (*P* < 5e-8); low linkage disequilibrium (LD) SNPs (LD r^2^ < 0.001, LD distance > 10,000kb); SNPs with an F-statistic exceeding 10, excluding the effect of the weak IV bias; (v) SNPs with palindromic sequences, as these SNPs failed to infer the orientation of their alleles and confound the MR estimation. Outcome-associated SNPs were removed when P_2_ < 5e-05. SNPs that were associated with confounders were excluded.

Preliminary MR analysis was conducted based on Inverse Variance Weighted (IVW), Mendelian randomization Egger (MR-Egger), Weighted Median (WM), and Weighted Mode (SNP ≥ 3). Without horizontal pleiotropy, the IVW method combines ratio estimates from each IV using meta-analytic techniques. Consequently, the results obtained using the IVW methodology serve as the main research findings.^[29]^ The MR Egger was primarily employed for MR causal inference when potential horizontal pleiotropy existed.^[30]^ The WM method is a statistical approach that requires more than 50% of the weights to originate from valid IVs, which is optimal in scenarios characterized by heterogeneity, but not horizontal pleiotropy.^[31]^ The weighted mode method has been shown to improve analytical outcomes by effectively accounting for discrepancies in genotype frequencies.^[32]^ When there were only 2 SNPs, the associations were analyzed using the IVW method only, whereas the Wald ratio method was used when only 1 SNP was available.^[33]^ In addition, re-analysis was performed following the removal of confounding factors, with FDR correlation performed to obtain more robust results.^[34]^ The threshold of the *P* value was *P* < .05, after FDR correlation.

Considering the direction of drug action on its target gene. For drugs that inhibit BP gene expression, the effect on disease risk was inferred as the inverse of the OR obtained from gene expression and outcome associations. Thus, OR < 1 for gene expression with disease corresponds to an increased risk associated with drug use.

Sensitivity analysis was performed, and no heterogeneity among the IVs was assumed when the *P*-value from Cochran Q test exceeded 0.05.^[29]^ Horizontal pleiotropy was also examined jointly using MR-Egger and MR-PRESSO tests, and no horizontal pleiotropy or outlier values were detected when *P* > .05.^[30,35]^ Leave-one-out (LOO) analysis involved the removal of individual SNPs one by one, followed by the analysis of the remaining data to ascertain the change in the overall effect.^[36]^ Moreover, the Steiger test was performed for each SNP to determine reverse causality.^[37]^ All MR analyses were performed using R software (version 4.4.2) using the “TwoSampleMR” package, and the P-values were adjusted with FDR correction.

We selected datasets without overlapping between each set of exposure and outcome.

### 2.9. Colocalization analysis

Shared causal variants between target genes and outcome diseases were investigated using co-localization analysis with 4 hypotheses.^[38]^ PPH4 values ≥ 0.8 were considered indicative of high-confidence colocalization. The study performed colocalization testing for BP and breast diseases, eQTL, and breast diseases. The analysis was implemented by “coloc” package of the R software.

### 2.10. Mediation analysis

Based on the literature, a potential link may exist among hypertension, vascular endothelium, and breast cancer.^[39]^ Data on vascular diseases were acquired from the UK Biobank for meditation analysis.^[40]^ Intestinal ischemia was then used as a mediator in a 2-step mediation analysis to partition the direct and indirect effects of BP, previously identified as a positive antihypertensive drug target, on breast diseases. The effect of SBP on intestinal ischemia was β1, the effect of intestinal ischemia on breast disease was β2, and the effect of SBP on breast disease was β0, which is also known as the total effect (TE). The indirect effect (i.e.) was determined as “β1×β2, with the proportion counted as “β1×β2/β0.”^[41]^ The delta method was also used to calculate confidence intervals (CIs) for the mediated proportions.^[42]^

The study used public data and no further ethic approval is needed.

## 3. Results

### 3.1. Identification of effective antihypertensive drug targets with CAD as positive control

**Figure 2.**
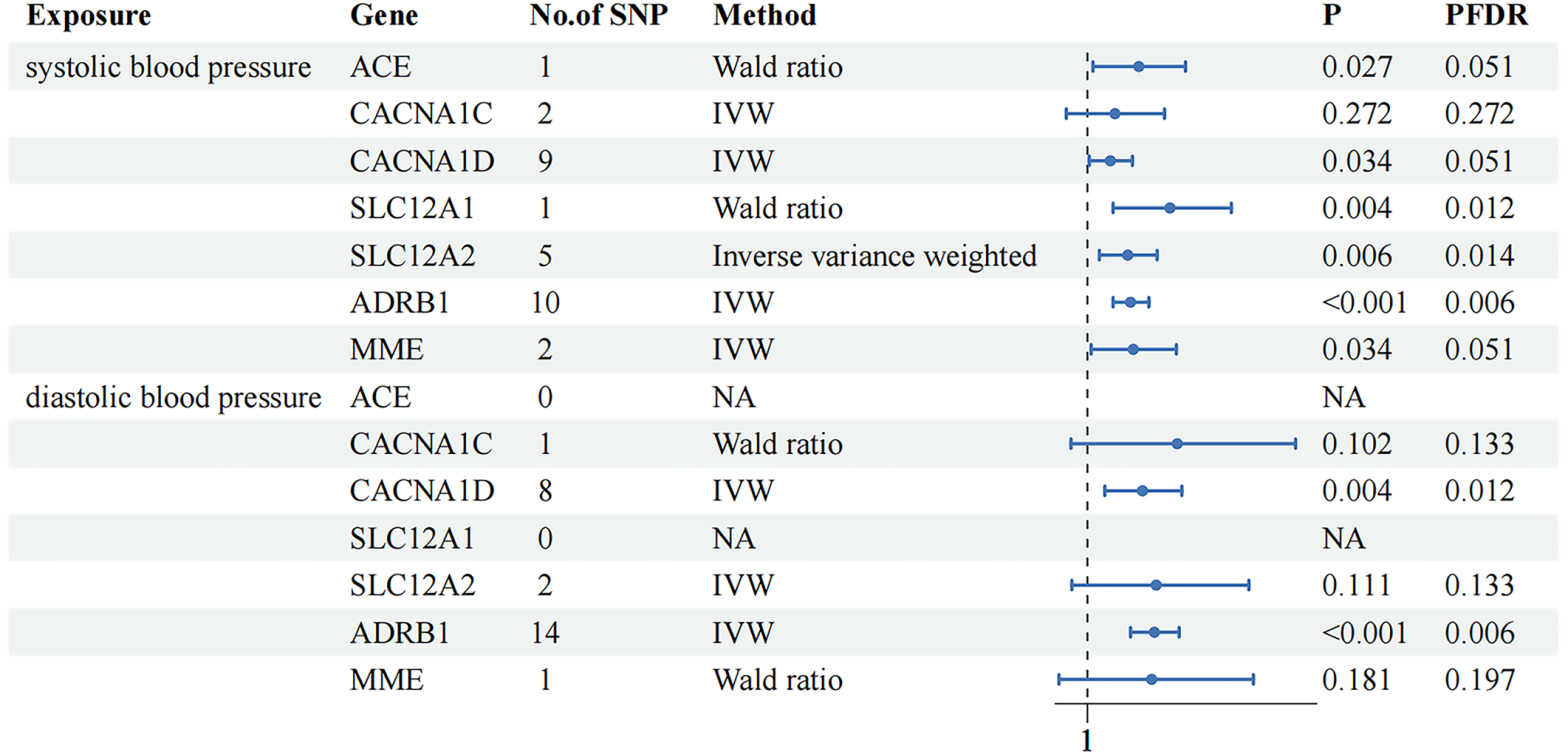
Associations between antihypertensive drugs’ genetic proxies and the risk of positive control (CAD) in European populations.

To assess the effectiveness of the selected antihypertensive drug targets (Table S3), we analyzed the causal effect of SBP and positive control outcome (CAD) in these gene loci. Three valid targets were identified across 2 antihypertensive drug groups (OR > 1, PFDR < 0.050) after FDR correction: loop diuretics (SLC12A1, SLC12A2) and BBs (ADRB1).^[43]^ Furthermore, the FDR-corrected results indicated that antihypertensive drugs targeting DBP may reduce CAD risk within the target of CACNA1D and ADRB1, as presented in Figure 2.

### 3.2. Relationship between antihypertensive drug targets and breast diseases

**Figure 3.**
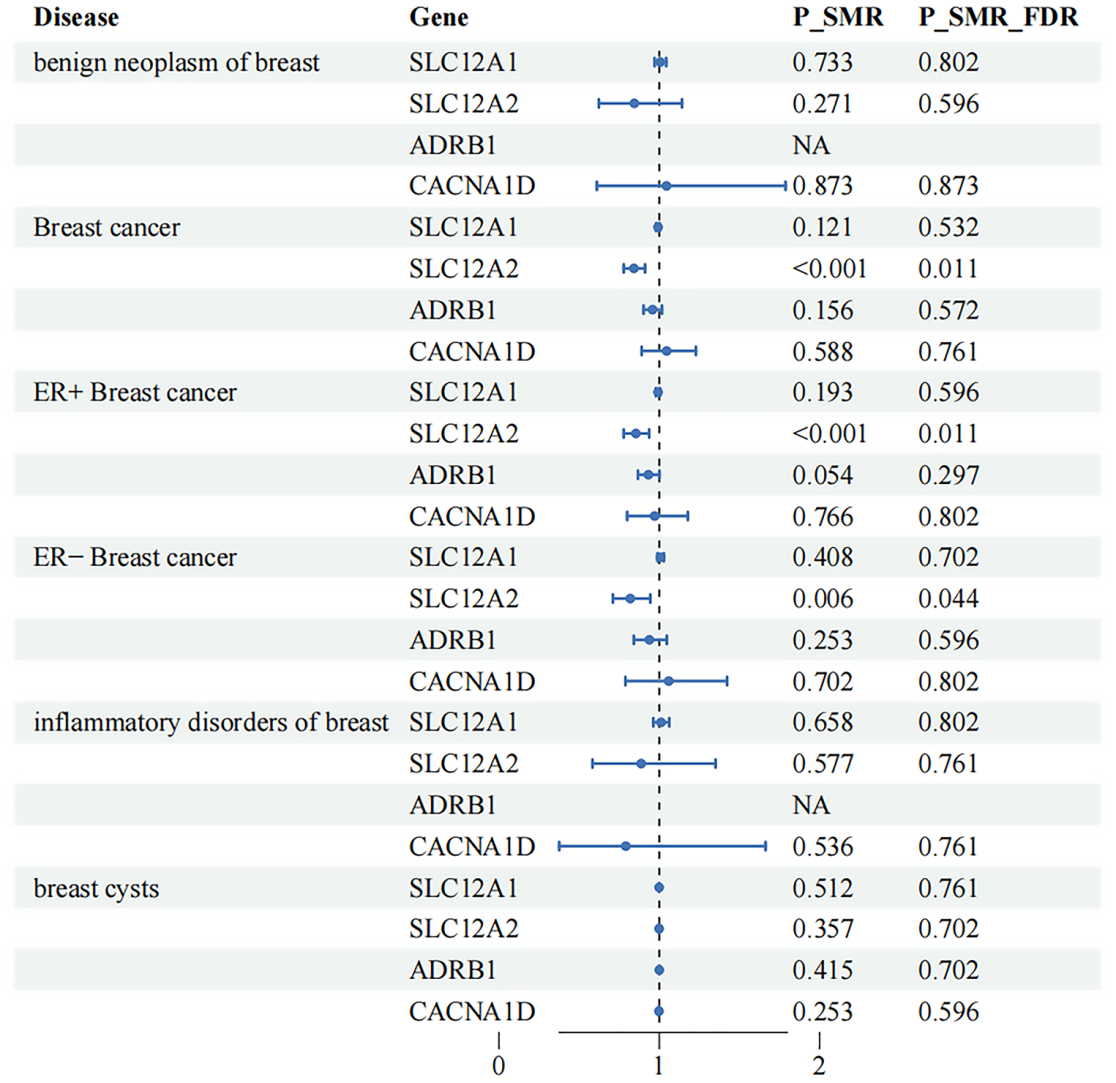
SMR analysis on the associations between the expression of 3 antihypertensive drug targeting genes in the blood (sourced from eQTLGen) with breast diseases. NA = no SNPs were extracted.

SMR analysis between blood-derived gene expression and breast diseases was performed on 3 drug target genes with clear therapeutic effects. The use of loop diuretics elevated the overall incidence of breast cancer by suppressing the SLC12A2 gene in the blood (OR_SMR = 0.842, 95% CI_SMR: 0.778–0.911, P_SMR = 1.87984e-05, P_SMR_FDR = 0.011). In addition, SMR analysis of the specific subtypes of breast cancer was performed. Loop diuretic drugs targeting SLC12A2 elevated the risk of estrogen receptor-positive breast cancer (ER + breast cancer) risk (OR_SMR: 0.854, 95% CI_SMR: 0.778–0.937, P_SMR = 0.0009, P_SMR_FDR = 0.011). Moreover, estrogen receptor-negetive breast cancer (ER-breast cancer) risk was increased by the use of loop diuretics targeting SLC12A2 (OR_SMR = 0.819, 95% CI_SMR: 0.711–0.944, P_SMR = 0.006, P_SMR_FDR = 0.044). All remaining results with P_HEIDI > 0.05 indicated that our results were not affected by heterogeneity, and all the findings are shown in Figure 3. The specific information of SNPs involved in SMR analysis is presented in Table S4.

### 3.3. Effects of genetically determined antihypertensive drug targets on breast diseases

Subsequently, a 2-sample MR analysis of genetically proxied BP and breast diseases was performed for the 3 selected genes. The specific information on SNPs involved in MR analysis between BP (SBP and DBP) and breast diseases is presented in Table S5. MR analysis showed that loop diuretics increased the overall breast cancer risk by reducing SBP and targeting SLC12A2 (OR = 0.964, 95% CI: 0.937–0.992, *P* = .012). Regarding the different subtypes of breast cancer, loop diuretics targeting SLC12A2 increased ER + breast cancer risk by mediating SBP (OR = 0.957, 95% CI: 0.925–0.990, *P* = .011), with support from the WM results (OR = 0.958; 95% CI, 0.918–0.999; *P* = .044). In addition, the IVW method suggested that loop diuretics targeting SLC12A2 also significantly increased the occurrence of ER-breast cancer by mediating SBP (OR = 0.948, 95% CI: 0.900–0.998, *P* = .042). Subsequently, FDR correction was applied, validating the increased incidence of overall (PFDR = 0.036) and ER + (PFDR = 0.033) breast cancer. Additionally, no direct association was found between common antihypertensive drug usage and the development of other breast diseases, and the details are shown in Table S6.

Via the downstream effects of DBP, antihypertensive drug was not associated with the occurrence of breast disease. Detailed findings regarding DBP and breast disease are presented in Table S7. The specific number of IVs at each stage of the analysis is listed in the flow diagram in Table S8.

### 3.4. Colocalization analysis

**Figure 4.**
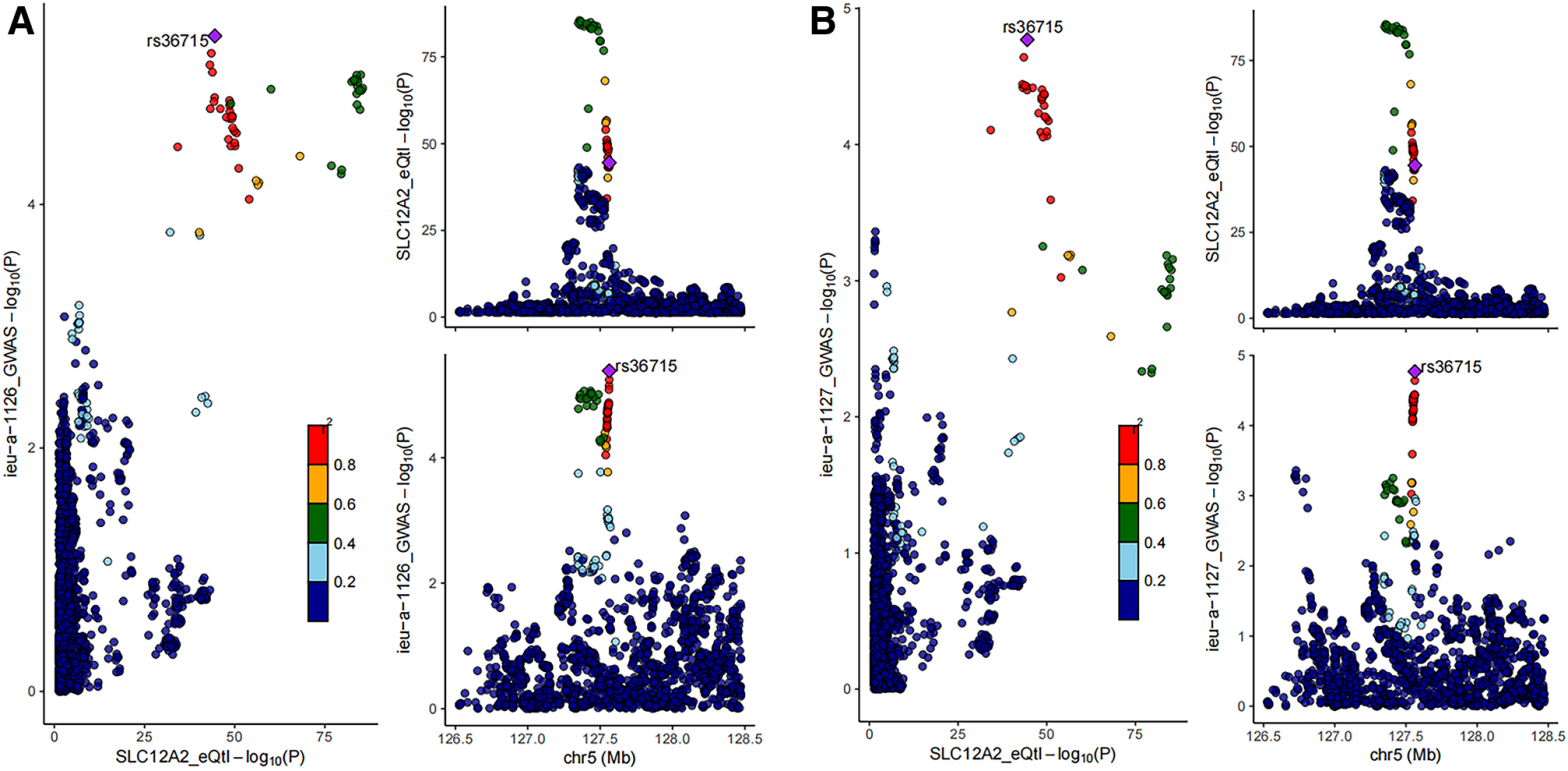
Colocalization analysis on causal association between SLC12A2 gene and breast cancer. (A) overall breast cancer, (B) ER + breast cancer.

Colocalization analysis was conducted to examine the connection between eQTL and overall/ER + breast cancer at the SNP level. Colocalization analysis provided strong support for a shared genetic variant between SLC12A2 expression in blood and overall breast cancer (PPH4.abf = 0.958). For ER + breast cancer, the analysis suggested a potential shared variant, although with weaker support (PPH4.abf = 0.612) (Fig. 4). Consistent with the SMR and MR results, these findings implicate SLC12A2 as a key gene through which loop diuretics may exert their effect on breast cancer risk. Therefore, SLC12A2, which is targeted by antihypertensive drugs, can promote breast cancer development.

Therefore, Consistent with the results obtained from the SMR analysis, the 2-sample MR analysis, and the colocalization analysis, loop diuretic drugs increased the risk of overall breast cancer and increased the risk of overall breast cancer partly, by targeting the SLC12A2 gene.

### 3.5. Mediation analysis

Through 2-step mediation analysis, intestinal ischemia was demonstrated to be a mediator between SLC12A2-targeted antihypertensive drugs and breast cancer. Further research should be conducted within the scope of SLC12A2, using SBP as a downstream mediator of antihypertensive drug effects. In the first step, MR analysis was used to determine the effective mediator (intestinal ischemia) and the effect of SBP on the mediator (β1). The second step was to perform an MR analysis, which validated the causality of the mediator in breast cancer (β2). The mediating effect sizes and their respective proportions of intestinal ischemia were analyzed for both overall and ER + breast cancers. The proportion of indirect effects of SBP on breast cancer mediated by intestinal ischemia was 0.737 (95% confidence interval [CI]: 0.129–1.345, *P* = .016). However, for ER + breast cancer, the proportion was 0.542 (95% CI: -0.010–1.093). The confidence intervals are wid and the results don’t have statistical significance. These findings suggest that intestinal ischemia mediates the effect of SBP on overall breast cancer within the SLC12A2 gene loci. Specific information on the SNPs involved in the mediation analysis is presented in Table S5.

## 4. Discussion

The study employed the drug-target and mediation MR analysis to investigate the long-term cumulative effect of antihypertensive drugs’ usage on the risk of developing breast diseases. Given that loop diuretics suppress SLC12A2 expression, our findings suggest that their long-term use may elevate breast cancer risk, consistent with the inverse of the observed ORs for gene expression. This study suggests a potential link that loop diuretics targeting SLC12A2 could elevate the risk of overall/ER + breast cancer by mediating SBP. Moreover, eQTLs shared common variant loci with the breast cancer-associated genes in SLC12A2. Subsequently, MR analysis demonstrated that the effect of SBP on overall breast cancer risk was partially mediated by intestinal ischemia. In contrast, common antihypertensive drug use did not have a direct effect on benign breast neoplasms, inflammatory breast disorders, and breast cysts yet. Therefore, significance should be attached to patients, who are using loop diuretics to manage hypertension, and health education regarding breast cancer risks and breast cancer screening should be strengthened. This provides guidance for primary and secondary prevention in breast cancer patients with hypertension history.

Gene expression modulated by loop diuretics was more significantly associated with breast cancer than other antihypertensive drugs, which is in line with the findings of many current observational studies. Previous studies suggested that loop diuretics were correlated with greater cancer risk but identified insignificant correlations with other hypertensive drugs.^[44]^ Su et al found that loop diuretic use was associated with an elevated risk of cutaneous squamous cell carcinoma,^[45]^ which was supported by another prospective study.^[46]^ Specifically, there are many related studies on loop diuretics and breast cancer. Largent et al found that patients with a history of hypertension and loop diuretic use were more susceptible to breast cancer.^[47]^ Chen et al comprehensively analyzed 14,766 patients with stage I/II breast cancer in the SEER database, suggesting that adverse outcomes were associated with the use of loop diuretics but not other classes of antihypertensive drugs.^[48]^ Wiranata et al found a significant correlation between loop diuretics and breast cancer in a meta-analysis.^[49]^ Alternative perspectives have also been proposed. Haibo et al found no significant association between antihypertensive drug use and breast cancer risk and demonstrated that long-term use of ACEIs and ARBs conferred protection against breast cancer development.^[50]^ Furthermore, large-scale retrospective cohort and database mining studies have found no significant correlation between antihypertensive medications and breast cancer.^[51,52]^

The mechanisms underlying these negative associations remain unclear. In terms of pharmacological mechanisms, these drugs inhibit the SLC12A family of Cl- transporters, thereby affecting the function of Na+-K + -2Cl- cotransporters (NKCC) 1 and 2. Loop diuretics also play a role in the release and binding of K+, which may induce a hyperkalemic state in the body.^[53]^ NKCC1 is located on the basolateral membrane of mammary epithelial cells and plays an important role in ductal epithelial cell maturation and mammary gland morphology.^[54]^ Wright et al found that downregulation of SLC12A2/NKCC1/BSC2 could affect vesicle trafficking and contribute to breast cancer cell formation.^[55]^ Another study found that NKCC activity was associated with a low membrane potential (MP) state, which is present in breast malignancies and implies impaired NKCC activity.^[55]^ However, different perspectives have been proposed. Khoshbakht et al suggested that SLC12A2 is significantly upregulated in breast cancer patients with brain metastases who would respond to correlated treatments.^[56]^

Some studies have confirmed that intestinal ischemia is associated with both antihypertensive drug use and breast cancer. In addition, ALLEN et al found through pathological screening that the use of K + and loop diuretics could lead to intestinal ischemia.^[57]^ A recent review article also suggested that vascular changes (including hypertension) would result in intestinal ischemia.^[58]^ Specifically, intestinal ischemia can disrupt the intestinal barrier, leading to an imbalance in the intestinal microbiota. Furthermore, intestinal microbiota imbalance can trigger cancer development by activating inflammasomes, promoting tumor growth, and facilitating angiogenesis.^[59]^ Additionally, intestinal microbiota contributes to the development of breast cancer through modulation of estrogen metabolism, immune status, and obesity.^[60]^ These findings were consistent with our finding that intestinal ischemia mediated the correlation between antihypertensive drugs and breast cancer.

This study had some limitations. First, as the IVs and breast diseases data exclusively comprised European participants, caution is warranted when applying these findings to diverse ethnic groups. Second, drug-targeted MR analysis assesses the lifetime cumulative effect of antihypertensive drugs rather than the short-term intervention. However, the timing of drug use can affect the onset of breast cancer. Therefore, the conclusion of the study only provides theoretical support for enhancing breast cancer screening in patients who have used loop diuretics for a long time. And it does not establish any harm associated with short-term use of loop diuretics. Third, the overall study design lacks validation through laboratory work. The association observed for loop diuretics, targeting SLC12A2, requires further in vitro or in vivo experiment to varify the target and clarify the signal pathway, leading to intestinal ischemia and increasing breast cancer risk.

Our study had some strengths. First, drug-target MR analysis identifies associations at the genetic level, providing more reliable results, because IVs are less susceptible to potential confounders. In addition, the robustness of the results was enhanced by combining the multiple MR methods. Second, compared to a previously published study, we used MR drug-targeting mediation analysis to comprehensively describe and summarize the association between antihypertensive drugs and breast diseases. Third, we used a drug-targeted mediation MR approach, which enabled the repositioning of drug targets and investigated possible mechanisms. Fourth, based on these studies, significance should be attached to breast cancer patients’ usage of loop diuretics to control blood pressure and to loop diuretic users’ breast cancer detection.

In this study, we comprehensively assessed the association between antihypertensive drugs and breast disease using a drug-targeted mediation MR framework with colocalization analysis. One possible interpretation of our data is that antihypertensives increased the risk of overall and ER + breast cancer through the SLC12A2 gene loci. And intestinal ischemia plays a mediating role between SBP and breast cnacer. However, no significant differences were observed yet between antihypertensive drug use and the incidence of other breast diseases. In conclusion, we suggest the effect of hypertensive drugs on breast disease, which provides information on the clinical management of breast disease. Our results may increase clinicians’ awareness regarding patients taking loop diuretics. It would be best if physicians strengthen breast cancer prevention and screening for them.

## Author contributions

**Conceptualization:** Jiaxuan Li, Kaili Zhang.

**Methodology:** Jiaxuan Li, Yiqun Ma.

**Writing – original draft:** Jiaxuan Li.

**Formal analysis:** Yiqun Ma.

**Software:** Yiqun Ma.

**Funding acquisition:** Kaili Zhang.

**Supervision:** Kaili Zhang.

**Writing – review & editing:** Kaili Zhang.
















