# Supplementary material for: Association of antihypertensive drugs, intestinal ischemia, and breast diseases: A drug-target Mendelian Randomization study
Source: Medicine (Baltimore). 2026 Jun 5;105(23):e49141. doi: 10.1097/MD.0000000000049141 (PMC13246127; doi:10.1097/MD.0000000000049141)
Supplement: Supplementary file 1 [file medi-105-e49141-s001.docx]

**STROBE-MR checklist of recommended items to address in reports of Mendelian randomization studies**^1^ ^2^

| **Item No.** | **Section** | **Checklist item** | **Page No.** | **Relevant text from manuscript** |
| --- | --- | --- | --- | --- |
| 1 | **TITLE and ABSTRACT** | Indicate Mendelian randomization (MR) as the study’s design in the title and/or the abstract if that is a main purpose of the study | 1 | Title: Association of antihypertensive drugs, intestinal ischemia, and breast diseases: A drug-target Mendelian Randomization study  Abstract: The study executed summary data-based Mendelian randomization (SMR) analysis of expression quantitative trait loci (eQTL) data in blood from patients with breast diseases. Then, blood pressure (BP) related single nucleotide polymorphisms (SNPs) were used as instrumental variables (IVs) to study the relationship between different antihypertensive drug targets and breast diseases, which was performed according to drug-targeting MR analysis. The data were extracted from FinnGen database, the BCAC database, the UK Biobank project, etc. False discovery rate (FDR) correction was executed for reliable results, while colocalization analysis was also performed to clarify shared gene variants. Furthermore, mediation MR methods were used to investigate the mechanistic pathways by which antihypertensives act on breast diseases in specific gene loci. |
|  | **INTRODUCTION** |  |  |  |
| 2 | **Background** | Explain the scientific background and rationale for the reported study. What is the exposure? Is a potential causal relationship between exposure and outcome plausible? Justify why MR is a helpful method to address the study question | 1-2 | Exposure: antihypertensive drugs  Yes, most studies promoted that hypertension is strongly correlated with heart failure, kidney disease and cancer. Because of high prevalence and mortality rates of the two illness, many studies are currently focusing on the relationship among hypertension, usage of antihypertensive drugs and breast diseases. Hypertension may promote cancer through systematic inflammatory process, modulation of associated cytokines, and alternation of cancer microenvironment. The relationship between antihypertensive drug usage and the risk of breast cancer have attracted the attention of more and more scholars, but the findings are not fully consistent. De Miranda et al. revealed that angiotensin-converting enzyme inhibitors (ACEIs) and angiotensin II receptor blockers (AREs) could inhibit breast cancer progression by suppressing metastasis, proliferation and angiogenesis. And a review including 57 articles also suggested that long-term use of ACEIs and ARBs was associated with reduced breast cancer risk, but use of calcium channel blockers (CCBs), beta blocker (BBs) and diuretics could increase breast cancer risk. Moreover, two case-control studies also demonstrated that CCBs, BBs, and diuretics increased the risk of breast cancer, but the role of ACEIs and ARBs were not significant. However, evidence from cross-sectional and cohort study have suggested the absence of significant relationship between antihypertensive medications (such as ACEIs, BBs, CCBs, and diuretics) and breast cancer development.  From our perspective, potential confounders have possibly led to conflicting results in previous observational studies. Furthermore, it is difficult to confirm causal relationships owing to the contribution of reverse causation. Furthermore, performing clinical drug trials are usually limited by funding constraints and ethical procedures. To fill this gap, a drug-target Mendelian randomization (MR) analysis was used provide more robust evidence of causality. MR analysis is founded on the random allocation of parental alleles to offspring, wherein the use of genetic variants as instrumental variables (IVs) effectively emulates the randomization process. This study used the drug target MR method. The potential implications for causal inference suggest that genetic variation within genes encoding drug targets contribute to modulation of drug targets activity and corresponding phenotypic outcomes. This inference is drawn using genome-wide association studies (GWAS) and expression quantitative trait loci (eQTL) data as proxies for exposure. So drug-targeted MR methods were performed to achieve more efficient drug development and remove translation barriers. The mediation MR method was also incorporated into the process. According to previous studies, blood pressure (BP) is strongly associated with altered vascular status, while breast cancer is also associated with ischemia. Therefore, we further investigated the causal relationship between BP, intestinal ischemia, and breast cancer through mediated MR analysis. |
| 3 | **Objectives** | State specific objectives clearly, including pre-specified causal hypotheses (if any). State that MR is a method that, under specific assumptions, intends to estimate causal effects | 2 | By using genetic proxies for drug targets, we conducted a drug-target MR study supplemented by colocalization analysis to figure out the correlation of various antihypertensive drugs and the onset of breast diseases. In addition, mediation analyses were performed from a genetic perspective on the causal relationship among hypertension, intestinal ischemia, and breast disease. |
|  | **METHODS** |  |  |  |
| 4 | **Study design and data sources** | Present key elements of the study design early in the article. Consider including a table listing sources of data for all phases of the study. For each data source contributing to the analysis, describe the following: |  |  |
|  | a) | Setting: Describe the study design and the underlying population, if possible. Describe the setting, locations, and relevant dates, including periods of recruitment, exposure, follow-up, and data collection, when available. | 2 | We utilized GWAS summary data and eQTLs data of European population. |
|  | b) | Participants: Give the eligibility criteria, and the sources and methods of selection of participants. Report the sample size, and whether any power or sample size calculations were carried out prior to the main analysis | 2-3 | 2.2 Identification of antihypertensive drug targets: According to widely accepted guidelines, hypertensive drugs are categorized as: ACEIs, ARBs, BBs, dihydropyridines‌, non-dihydropyridines, thiazide diuretics (TDs), loop diuretics, potassium-sparing diuretics, renin inhibitors, mineralocorticoid receptor antagonists, alpha-1 blockers, centrally acting drugs, vasodilators, and angiotensin receptor-neprilysin inhibitors. Subsequently, the shared gene targets of the 14 antihypertensive drugs have been identified in the DrugBank (https://go.drugbank.com/) and the ChEMBL (https://www.ebi.ac.uk/chembl/). Then, we retrieved the details of these genes in the NCBI Gene database (https://www.ncbi.nlm.nih.gov/), which were listed in Supplementary Table 2.  2.3 Identification of BP: In this study, downstream effects of pharmacological interventions (e.g. BP) were selected, to model drug exposure. Genetic association data for SBP were derived from the IEU Open GWAS Project database (https://gwas.mrcieu.ac.uk/, Dataset: ieu-b-38). The present study encompassed 757,601 European pedigree individuals from the UK Biobank and the International Consortium of Blood Pressure. Meanwhile, data on diastolic blood pressure (DBP) from the same population was also obtained (Dataset: ieu-b-39), and all participants were adjusted in age, sex, and BMI.  2.4 Identification of gene expression data in the blood: Currently, it is widely accepted that peripheral blood is readily accessible for detection, and the eQTL effects in peripheral blood may reflect of those in other relevant tissues. As a result, eQTL data was obtained, which encompassed a total of 31,684 blood samples from individuals of European descent and provided transcriptomic profiles of 16,987 genes. Association analysis was also used to identify loci of variation linked to gene expression. In addition, aggregated data for the eQTL were extracted from the eQTLGen consortium (https://www. eqtlgen.org/).  2.5 Determination of study outcomes: Summary-level GWAS data related to benign breast neoplasm and inflammatory breast disorders were available in the FinnGen study (https://www.finngen.fi/en). Summary-level data of breast cancer, which combined GWAS meta-analysis data, iCOGS and Oncoarray, were obtained from the Breast Cancer Association Consortium (BCAC) database (https://bcac.ccge.medschl.cam.ac.uk). And data of breast cysts were selected from the UK Biobank project (http://www.nealelab.is/uk-biobank/). All of participants were European population and no overlap existed between the populations in the exposure and outcome datasets. Details on specific datasets is presented in Supplementary Table 3.  2.6 Positive control: To verify the validity of genetic targets, positive control analysis was performed. Antihypertensive drugs have been widely utilized to manage coronary artery disease (CAD), so we obtained the dataset of CAD from the GWAS catalog database (https://www.ebi.ac.uk/gwas/home) for analysis (Dataset: ebi-a-GCST005195). This is the largest dataset for the study of CAD with 122,733 cases and 424,528 controls. In this process, two-sample MR analysis was completed within the scope of the target gene identified previously.  Details on specific datasets is presented in Supplementary Table 3. |
|  | c) | Describe measurement, quality control and selection of genetic variants | 3 | SMR analysis: SMR analysis was conducted to ascertain the genetic impact of eQTL, which utilized summary data and followed the principles of MR. Horizontal pleiotropy was assessed by the Heterogeneity in dependent instrument (HEIDI) method. The threshold of P value was 0.05 after FDR correlation.  Two-sample MR analysis: We performed positive control MR analysis, with SBP and DBP as exposure, CAD as outcome. Then, we conducted the two-sample MR analysis, with SBP and DBP as exposure, and breast diseases as outcome. Firstly, we selected IVs with following screening criteria: (i) select 1,000kb gene region cis-SNPs located near the drug target genes; (ii) select SNPs strongly associated with BP (P<5e-8); (iii) select low linkage disequilibrium (LD) SNPs (LD r2<0.001, LD distance>10,000kb); (iv) select SNPs with an F-statistic exceeding 10, excluding the effect of the weak IV bias; (v) exclude SNPs with palindromic sequences, as these SNPs fail to infer the orientation of their alleles and confound the MR estimation. |
|  | d) | For each exposure, outcome, and other relevant variables, describe methods of assessment and diagnostic criteria for diseases | 3 | SMR analysis: The threshold of P value was 0.05 after FDR correlation.  Two-sample MR analysis: The threshold of P value was P < 0.05 after FDR correlation. |
|  | e) | Provide details of ethics committee approval and participant informed consent, if relevant | 4 | The study used public data and no further ethic approval is needed. |
| 5 | **Assumptions** | Explicitly state the three core IV assumptions for the main analysis (relevance, independence and exclusion restriction) as well assumptions for any additional or sensitivity analysis | 2-3 | The validity of a two-sample MR study is contingent upon three basic assumptions: (i) Association: the IVs selected from the data demonstrates a strong correlation with the exposure; (ii) Independence: the IVs have been shown not to be linked to any confounders of the exposure; and (iii) Exclusivity: the IVs is correlated with the outcome solely through the exposure.  SMR analysis: Horizontal pleiotropy was assessed by the Heterogeneity in dependent instrument (HEIDI) method.  Two-sample MR analysis: Sensitivity analysis was performed, and no heterogeneity among the IVs was assumed when the P-value from Cochran’s Q test exceeded 0.05 . Horizontal pleiotropy was also examined jointly by MR-Egger and MR-PRESSO test, and no horizontal pleiotropy or outlier value were detected when P > 0.05 . Leave-one-out (LOO) analysis involved the removal of individual SNPs one by one, followed by the analysis of the remaining data to ascertain the change of overall effect. Moreover, Steiger test was performed on each SNP for reverse causality. |
| 6 | **Statistical methods: main analysis** | Describe statistical methods and statistics used |  |  |
|  | a) | Describe how quantitative variables were handled in the analyses (i.e., scale, units, model) | 2-3 | Hypertensive drugs are categorized as: ACEIs, ARBs, BBs, dihydropyridines‌, non-dihydropyridines, thiazide diuretics (TDs), loop diuretics, potassium-sparing diuretics, renin inhibitors, mineralocorticoid receptor antagonists, alpha-1 blockers, centrally acting drugs, vasodilators, and angiotensin receptor-neprilysin inhibitors. Details were listed in Supplementary Table 2.  We performed positive control MR analysis, with SBP and DBP as exposure, CAD as outcome.  Breast diseases mainly encompassed benign breast neoplasms, inflammatory breast diseases, and breast cysts. |
|  | b) | Describe how genetic variants were handled in the analyses and, if applicable, how their weights were selected | 3 | We selected IVs with following screening criteria: (i) select 1,000kb gene region cis-SNPs located near the drug target genes; (ii) select SNPs strongly associated with BP (P<5e-8); (iii) select low linkage disequilibrium (LD) SNPs (LD r2<0.001, LD distance>10,000kb); (iv) select SNPs with an F-statistic exceeding 10, excluding the effect of the weak IV bias; (v) exclude SNPs with palindromic sequences, as these SNPs fail to infer the orientation of their alleles and confound the MR estimation.  The shared gene targets of the 14 antihypertensive drugs have been identified in the DrugBank (https://go.drugbank.com/) and the ChEMBL (https://www.ebi.ac.uk/chembl/). Then, we retrieved the details of these genes in the NCBI Gene database (https://www.ncbi.nlm.nih.gov/), which were listed in Supplementary Table 2. |
|  | c) | Describe the MR estimator (e.g. two-stage least squares, Wald ratio) and related statistics. Detail the included covariates and, in case of two-sample MR, whether the same covariate set was used for adjustment in the two samples | 3 | SMR analysis: SMR analysis was conducted to ascertain the genetic impact of eQTL, which utilized summary data and followed the principles of MR.  Two-sample MR analysis: Preliminary MR analysis was conducted based on Inverse Variance Weighted (IVW), along with Mendelian randomization Egger (MR-Egger), Weighted Median (WM) and Weighted Mode (SNP≥3). In essence, without horizontal pleiotropy, IVW method combined ratio estimates from each IV using meta-analytic techniques. As a consequence, the results obtained by the IVW methodology served as the main research findings. MR Egger was primarily employed for MR causal inference when potential horizontal pleiotropy existed. The WM method is a statistical approach that necessitates more than 50% of the weights to originate from valid IVs, which is optimal in scenarios characterized by heterogeneity but not horizontal pleiotropy. The weighted mode method has been shown to improve analytic outcomes by effectively accounting for discrepancies in genotype frequencies. When there were only 2 SNPs, the associations were analyzed by IVW method only, while Wald ratio method were used when only one SNP was available. In addition, re-analysis was done following the removal of confounding factors, with FDR correlation performed to obtain more robust results.  Mediation analysis: The effect of SBP on intestinal ischemia was β1, the effect of intestinal ischemia on breast diseases was β2, and the effect of SBP on breast diseases was β0, which was also known as total effect (TE). Indirect effect (IE) was determined as “β1×β2”, with its proportion counted as “β1×β2/β0”. The delta method was also used to calculate the confidence intervals (CIs) for the mediated proportions. |
|  | d) | Explain how missing data were addressed | 4-5 | In Fig2, Fig3, Fig4, if there is missing data, wefilled it with “NA”. |
|  | e) | If applicable, indicate how multiple testing was addressed | 5 | SMR analysis: Horizontal pleiotropy was assessed by the Heterogeneity in dependent instrument (HEIDI) method.  Two-sample MR analysis: Sensitivity analysis was performed, and no heterogeneity among the IVs was assumed when the P-value from Cochran’s Q test exceeded 0.05 . Horizontal pleiotropy was also examined jointly by MR-Egger and MR-PRESSO test, and no horizontal pleiotropy or outlier value were detected when P > 0.05 . Leave-one-out (LOO) analysis involved the removal of individual SNPs one by one, followed by the analysis of the remaining data to ascertain the change of overall effect. Moreover, Steiger test was performed on each SNP for reverse causality. The P-values were adjusted with FDR correction. The threshold of P value was P < 0.05 after FDR correlation. |
| 7 | **Assessment of assumptions** | Describe any methods or prior knowledge used to assess the assumptions or justify their validity | 3 | SMR analysis: SMR analysis was conducted to ascertain the genetic impact of eQTL, which utilized summary data and followed the principles of MR.  Two-sample MR analysis: MR analysis was conducted based on Inverse Variance Weighted (IVW), along with Mendelian randomization Egger (MR-Egger), Weighted Median (WM) and Weighted Mode (SNP≥3). In essence, without horizontal pleiotropy, IVW method combined ratio estimates from each IV using meta-analytic techniques. As a consequence, the results obtained by the IVW methodology served as the main research findings. MR Egger was primarily employed for MR causal inference when potential horizontal pleiotropy existed. The WM method is a statistical approach that necessitates more than 50% of the weights to originate from valid IVs, which is optimal in scenarios characterized by heterogeneity but not horizontal pleiotropy. The weighted mode method has been shown to improve analytic outcomes by effectively accounting for discrepancies in genotype frequencies. When there were only 2 SNPs, the associations were analyzed by IVW method only, while Wald ratio method were used when only one SNP was available. |
| 8 | **Sensitivity analyses and additional analyses** | Describe any sensitivity analyses or additional analyses performed (e.g. comparison of effect estimates from different approaches, independent replication, bias analytic techniques, validation of instruments, simulations) | 3, 4 | Positive control: To verify the validity of genetic targets, positive control analysis was performed. Antihypertensive drugs have been widely utilized to manage coronary artery disease (CAD).  SMR analysis: Horizontal pleiotropy was assessed by the Heterogeneity in dependent instrument (HEIDI) method. The threshold of P value was 0.05 after FDR correlation.  Two-sample MR analysis: Sensitivity analysis was performed, and no heterogeneity among the IVs was assumed when the P-value from Cochran’s Q test exceeded 0.05 . Horizontal pleiotropy was also examined jointly by MR-Egger and MR-PRESSO test, and no horizontal pleiotropy or outlier value were detected when P > 0.05 . Leave-one-out (LOO) analysis involved the removal of individual SNPs one by one, followed by the analysis of the remaining data to ascertain the change of overall effect. Moreover, Steiger test was performed on each SNP for reverse causality.The P-values were adjusted with FDR correction. The threshold of P value was P < 0.05 after FDR correlation.  Colocalization analysis: Shared causal variants between target genes and outcome diseases were investigated using colocalization analysis, with four hypotheses. PPH4 values of ≥ 0.8 were considered indicative of high-confidence colocalization. The study performed colocalization testing for BP and breast diseases, eQTL and breast diseases, respectively. |
| 9 | **Software and pre-registration** |  |  |  |
|  | a) | Name statistical software and package(s), including version and settings used | 3,4 | All the MR analysis were done in R software (version 4.4.2) using the “TwoSampleMR” package.  The analysis was implemented by “coloc” package of the R software. |
|  | b) | State whether the study protocol and details were pre-registered (as well as when and where) |  | No. The study used public data, so the study protocol and details didn’t need to be pre-registered. |
|  | **RESULTS** |  |  |  |
| 10 | **Descriptive data** |  |  |  |
|  | a) | Report the numbers of individuals at each stage of included studies and reasons for exclusion. Consider use of a flow diagram | 5 | The specific numbers of IVs at each stage of the analysis is listed in a flow diagram in Supplementary Table S8. |
|  | b) | Report summary statistics for phenotypic exposure(s), outcome(s), and other relevant variables (e.g. means, SDs, proportions) | 4,5 | SMR analysis: The specific information of SNPs, which are involved in SMR analysis, is presented in Supplementary Table S4.  Two-sample MR analysis: The specific information of SNPs, involved in MR analysis between BP (SBP and DBP) and breast diseases, is presented in Supplementary Table S5.  Mediation analysis: The specific information of SNPs, involved in mediation analysis is presented in Supplementary Table S5. |
|  | c) | If the data sources include meta-analyses of previous studies, provide the assessments of heterogeneity across these studies | 4-5 | SMR analysis: All remaining results with P_HEIDI > 0.05 indicated that our results were not affected by heterogeneity.  Two-sample MR analysis: Among these associations, none of them presented heterogeneity, and the MR-Egger intercept suggested that none of the interrelationships were affected by horizontal pleiotropy. According to the MR-PRESSO test, no outliers was found. All analyses passed The Steiger test with no reverse causality existed. |
|  | d) | For two-sample MR:  i.  Provide justification of the similarity of the genetic variant-exposure associations between the exposure and outcome samples  ii.  Provide information on the number of individuals who overlap between the exposure and outcome studies | 3 | Select SNPs strongly associated with BP (P<5e-8); select SNPs with an F-statistic exceeding 10, excluding the effect of the weak IV bias.  Without overlapping between each set of exposure and outcome. |
| 11 | **Main results** |  |  |  |
|  | a) | Report the associations between genetic variant and exposure, and between genetic variant and outcome, preferably on an interpretable scale | 3 | We conducted the two-sample MR analysis, with SBP and DBP as exposure, and breast diseases as outcome. Firstly, we selected IVs with following screening criteria: (i) select 1,000kb gene region cis-SNPs located near the drug target genes; (ii) select SNPs strongly associated with BP (P<5e-8); (iii) select low linkage disequilibrium (LD) SNPs (LD r2<0.001, LD distance>10,000kb); (iv) select SNPs with an F-statistic exceeding 10, excluding the effect of the weak IV bias; (v) exclude SNPs with palindromic sequences, as these SNPs fail to infer the orientation of their alleles and confound the MR estimation.  While outcome-associated SNPs were removed when P2<5e-05. Moreover, SNPs associated with confounders were also excluded. |
|  | b) | Report MR estimates of the relationship between exposure and outcome, and the measures of uncertainty from the MR analysis, on an interpretable scale, such as odds ratio or relative risk per SD difference | 4,5 | SMR analysis: The usage of loop diuretics elevated the incidence of overall breast cancer by suppressing the SLC12A2 gene in the blood (OR_SMR = 0.842, 95% CI_SMR: 0.778-0.911, P_SMR = 1.87984e-05, P_SMR_FDR = 0.011). In addition, SMR analysis on specific subtypes of breast cancer was completed. Loop diuretics drugs targeting SLC12A2 elevated ER+ breast cancer risk (OR_SMR = 0.854, 95% CI_SMR: 0.778-0.937, P_SMR = 0.0009, P_SMR_FDR = 0.011). Moreover, ER- breast cancer risk was increased by the usage of loop diuretics targeting SLC12A2 (OR_SMR = 0.819, 95% CI_SMR: 0.711-0.944, P_SMR = 0.006, P_SMR_FDR = 0.044).  Two-sample MR analysis: In terms of the relationship between SBP and breast diseases, MR analysis showed that loop diuretics increased overall breast cancer risk by reducing SBP and targeting SLC12A2 (OR = 0.964, 95% CI: 0.937–0.992, P = 0.012), as supported by WM method (P = 0.039). As for different subtypes of breast cancer, loop diuretics targeting SLC12A2 increased ER+ breast cancer risk by mediating SBP (OR = 0.957, 95% CI: 0.925 – 0.990, P = 0.011), with supports from WM results (OR = 0.958, 95% CI: 0.918 – 0.999, P = 0.044). In addition, the IVW method suggested that loop diuretics targeting SLC12A2 also significantly increased the occurrence of ER- breast cancer by mediating SBP (OR = 0.948, 95% CI: 0.900 –0.998, P = 0.042). Subsequently, FDR correction was applied, validating the increased incidence of overall (P = 0.036) and ER+ (P = 0.033) breast cancer. But no analogous results were identified by MR-Egger, WM, and Weighted mode methods. Additionally, no direct association was found between common antihypertensive drug usage and the development of other breast diseases, and the details are shown in Supplementary Table 4. With regard to the association between DBP and breast diseases identified by IVW method, DBP was linked to elevated risk of breast cancer within the ADRB1 gene, which was targeted by beta-blockers (OR = 0.968, 95% CI: 0.941 – 0.994, P = 0.018).  Mediation analysis: The proportion of indirect effects of SBP on breast cancer mediated by intestinal ischemia was 0.737 (95% CI: 0.129 ~ 1.345, P = 0.016). However, for ER+ breast cancer, the proportion was 0.542 (95% CI: -0.010 ~ 1.093) without statistical significance. |
|  | c) | If relevant, consider translating estimates of relative risk into absolute risk for a meaningful time period |  | Not applicable |
|  | d) | Consider plots to visualize results (e.g. forest plot, scatterplot of associations between genetic variants and outcome versus between genetic variants and exposure) | 4,5 | Fig 2 Associations between antihypertensive drugs’ genetic proxies and the risk of positive control (CAD) in European populations.  Fig 3 SMR analysis on the associations between the expression of three antihypertensive drug targeting genes in the blood (sourced from eQTLGen) with breast diseases.  Fig 4 Colocalization analysis on causal association between SLC12A2 gene and breast cancer. (i) overall breast cancer, (ii) ER+ breast cancer. |
| 12 | **Assessment of assumptions** |  |  |  |
|  | a) | Report the assessment of the validity of the assumptions | 3 | SMR analysis: SMR analysis was conducted to ascertain the genetic impact of eQTL, which utilized summary data and followed the principles of MR. Horizontal pleiotropy was assessed by the Heterogeneity in dependent instrument (HEIDI) method. The threshold of P value was 0.05 after FDR correlation.  Two-sample MR analysis: Sensitivity analysis was performed, and no heterogeneity among the IVs was assumed when the P-value from Cochran’s Q test exceeded 0.05 . Horizontal pleiotropy was also examined jointly by MR-Egger and MR-PRESSO test, and no horizontal pleiotropy or outlier value were detected when P > 0.05 . Leave-one-out (LOO) analysis involved the removal of individual SNPs one by one, followed by the analysis of the remaining data to ascertain the change of overall effect. Moreover, Steiger test was performed on each SNP for reverse causality. |
|  | b) | Report any additional statistics (e.g., assessments of heterogeneity across genetic variants, such as *I^2^*, Q statistic or E-value) | 4,5 | SMR analysis: All remaining results with P_HEIDI > 0.05 indicated that our results were not affected by heterogeneity, and all the findings were shown in Fig 3.  Two-sample MR analysis: Among these associations, none of them presented heterogeneity, and the MR-Egger intercept suggested that none of the interrelationships were affected by horizontal pleiotropy. According to the MR-PRESSO test, no outliers was found. All analyses passed The Steiger test with no reverse causality existed. |
| 13 | **Sensitivity analyses and additional analyses** |  |  |  |
|  | a) | Report any sensitivity analyses to assess the robustness of the main results to violations of the assumptions | 4,5 | SMR analysis: All remaining results with P_HEIDI > 0.05 indicated that our results were not affected by heterogeneity, and all the findings were shown in Fig 3.  Two-sample MR analysis: Among these associations, none of them presented heterogeneity, and the MR-Egger intercept suggested that none of the interrelationships were affected by horizontal pleiotropy. According to the MR-PRESSO test, no outliers was found. All analyses passed The Steiger test with no reverse causality existed. |
|  | b) | Report results from other sensitivity analyses or additional analyses | 5 | Colocalization analysis: Colocalization analysis was conducted to examine the connection of eQTL and overall/ER+ breast cancer at SNP level. We found that SLC12A2 exhibited significant shared genetic loci between blood and overall breast cancer (PPH4.abf = 0.958). In addition, SLC12A2 in blood and ER+ breast cancer shared the same variant to a certain extent (PPH4.abf = 0.612).  Mediation analysis: The proportion of indirect effects of SBP on breast cancer mediated by intestinal ischemia was 0.737 (95% CI: 0.129 ~ 1.345, P = 0.016). However, for ER+ breast cancer, the proportion was 0.542 (95% CI: -0.010 ~ 1.093) without statistical significance. |
|  | c) | Report any assessment of direction of causal relationship (e.g., bidirectional MR) | 5 | All analyses passed The Steiger test with no reverse causality existed. |
|  | d) | When relevant, report and compare with estimates from non-MR analyses | 5,6 | Gene expression modulated by loop diuretics was more significantly associated with breast cancer compare to other antihypertensive drugs, which was in line with the findings of many current observational studies. Previous studies suggested that loop diuretics was correlated with greater cancer risk, but identified insignificant correlation of other hypertensive drugs. |
|  | e) | Consider additional plots to visualize results (e.g., leave-one-out analyses) | 5 | Fig 4 Colocalization analysis on causal association between SLC12A2 gene and breast cancer. (i) overall breast cancer, (ii) ER+ breast cancer. |
|  | **DISCUSSION** |  |  |  |
| 14 | **Key results** | Summarize key results with reference to study objectives | 5 | This study suggested that loop diuretics targeting SLC12A2 could elevate the risk of overall/ER+ breast cancer by mediating SBP. Moreover, eQTLs shared common variant loci with breast cancer-associated genes within SLC12A2. Subsequently, mediated MR analysis demonstrated that the effect of SBP on breast cancer risk was partially mediated by intestinal ischemia. In contrast, there was no direct effect of common antihypertensive drug usage on benign breast neoplasm, inflammatory breast disorders and breast cysts. |
| 15 | **Limitations** | Discuss limitations of the study, taking into account the validity of the IV assumptions, other sources of potential bias, and imprecision. Discuss both direction and magnitude of any potential bias and any efforts to address them | 6 | This study has some limitations. First, as the study population comprised exclusively European participants, caution is warranted when applying these findings to diverse ethnic groups. Second, drug-targeted MR analysis assesses the lifetime cumulative effect of a drug rather than short-term intervention. However, the timing of drug use can affect the onset of breast cancer, which should be considered in future investigations. Also, the overall study design lacks validation from lab work. |
| 16 | **Interpretation** |  |  |  |
|  | a) | Meaning: Give a cautious overall interpretation of results in the context of their limitations and in comparison with other studies | 6 | In this study, we comprehensively assessed the association between antihypertensive drugs and breast diseases using a drug-targeted mediation MR framework with colocalization analysis. The results showed that antihypertensives increased the risk of overall and ER+ breast cancer through SLC12A2 gene loci, with intestinal ischemia playing a meditating role. However, no significant results were presented between antihypertensive drug use and other breast diseases. In conclusion, we suggested the effect of hypertensive drugs on breast diseases, which provided information on the clinical management of breast diseases. |
|  | b) | Mechanism: Discuss underlying biological mechanisms that could drive a potential causal relationship between the investigated exposure and the outcome, and whether the gene-environment equivalence assumption is reasonable. Use causal language carefully, clarifying that IV estimates may provide causal effects only under certain assumptions | 8-9 | The mechanisms of negative associations remain to be explored. In terms of pharmacological mechanisms, these drugs inhibit the SLC12A family of Cl- transporters, thereby affecting the function of Na+-K+-2Cl- cotransporter (NKCC) 1 and 2. Loop diuretics also play a role in the release and binding of K+, which may induce a hyperkalemic state in the body. NKCC1 protein are located on the basolateral membrane of mammary epithelial cells, playing an important role in ductal epithelial cell maturation and mammary gland morphology. Wright et al. found that down-regulation of SLC12A2/NKCC1/BSC2 could affect vesicle trafficking and contribute to breast cancer cell formation. Another study found that NKCC activity was associated with a low membrane potential (MP) state, which was presented in breast malignancies and implied impaired NKCC activity. However, there are also different perspectives. Khoshbakht et al. suggested that the SLC12A2 gene was significantly upregulated among breast cancer patients with brain metastases, who would respond to correlated treatments.  Currently, some studies have confirmed that intestinal ischemia is associated with both antihypertensive drugs and breast cancer. In addition, ALLEN et al. found through pathological screening that the use of K+ and loop diuretics could lead to intestinal ischemia. A recent review article also suggested that vascular changes (including hypertension) would result in intestinal ischemia. Specifically, intestinal ischemia could cause intestinal barrier disruption, leading to imbalance of intestinal microbiota. Furthermore, intestinal microbiota imbalance could trigger cancer development by activating inflammasomes, promoting tumor growth, and facilitating angiogenesis. Additionally, intestinal microbiota contributed to the development of breast cancer through modulation of estrogen metabolism, immune status, obesity, etc. These findings were consistent with our findings that intestinal ischemia mediated the correlation of antihypertensive drugs and breast cancer. |
|  | c) | Clinical relevance: Discuss whether the results have clinical or public policy relevance, and to what extent they inform effect sizes of possible interventions | 6 | Forth, based on these study, significance should be attached to breast cancer patients’ usage of loop diuretics to contral blood pressure, and loop diuretics users’ breast cancer detection. |
| 17 | **Generalizability** | Discuss the generalizability of the study results (a) to other populations, (b) across other exposure periods/timings, and (c) across other levels of exposure | 6 | As the study population comprised exclusively European participants, caution is warranted when applying these findings to diverse ethnic groups.  Drug-targeted MR analysis assesses the lifetime cumulative effect of a drug rather than short-term intervention. |
|  | **OTHER INFORMATION** |  |  |  |
| 18 | **Funding** | Describe sources of funding and the role of funders in the present study and, if applicable, sources of funding for the databases and original study or studies on which the present study is based | 7 | The research leading to these results received funding from the 2023 Hebei Provincial Medical Science Research Subjects Program, under Grant Agreement No.20230638. |
| 19 | **Data and data sharing** | Provide the data used to perform all analyses or report where and how the data can be accessed, and reference these sources in the article. Provide the statistical code needed to reproduce the results in the article, or report whether the code is publicly accessible and if so, where | 2-3, 6-7 | Availability of data and materials：  the DrugBank: https://go.drugbank.com/  the ChEMBL: https://www.ebi.ac.uk/chembl/  the NCBI Gene database: https://www.ncbi.nlm.nih.gov/  the IEU Open GWAS Project database: https://gwas.mrcieu.ac.uk/  the eQTLGen consortium: https://www. eqtlgen.org/  the FinnGen study: https://www.finngen.fi/en  the Breast Cancer Association Consortium database: https://bcac.ccge.medschl.cam.ac.uk  the UK Biobank project: http://www.nealelab.is/uk-biobank/  the GWAS catalog database: https://www.ebi.ac.uk/gwas/home  This study obtained GWAS data from public databases and all data is publicly available and accessible. |
| 20 | **Conflicts of Interest** | All authors should declare all potential conflicts of interest | 7 | The authors have no competing interests to declare that are relevant to the content of this article. |

This checklist is copyrighted by the Equator Network under the Creative Commons Attribution 3.0 Unported (CC BY 3.0) license.

1. Skrivankova VW, Richmond RC, Woolf BAR, Yarmolinsky J, Davies NM, Swanson SA, et al. Strengthening the Reporting of Observational Studies in Epidemiology using Mendelian Randomization (STROBE-MR) Statement. JAMA. 2021;under review.

2. Skrivankova VW, Richmond RC, Woolf BAR, Davies NM, Swanson SA, VanderWeele TJ, et al. Strengthening the Reporting of Observational Studies in Epidemiology using Mendelian Randomisation (STROBE-MR): Explanation and Elaboration. BMJ. 2021;375:n2233.
